# Validation of the PAM-13 instrument in the Hungarian general population 40 years old and above

**DOI:** 10.1007/s10198-022-01434-0

**Published:** 2022-01-31

**Authors:** Zsombor Zrubka, Péter Vékás, Péter Németh, Ágota Dobos, Ottó Hajdu, Levente Kovács, László Gulácsi, Judith Hibbard, Márta Péntek

**Affiliations:** 1 University Research and Innovation Center, Óbuda University, Bécsi út 96/b, Budapest, 1034 Hungary; 2 Corvinus Institute for Advanced Studies, Corvinus University of Budapest, Fővám tér 8, Budapest, 1093 Hungary; 3 Institute of Mathematical Statistics and Modelling, Corvinus University of Budapest, Fővám tér 8, Budapest, 1093 Hungary; 4 Doctoral School of Economics, Business and Informatics, Corvinus University of Budapest, Fővám tér 8, Budapest, 1093 Hungary; 5 Centre for Foreign Language Education and Research, Corvinus University of Budapest, Fővám tér 8, Budapest, 1093 Hungary; 6 Institute of Business Economics, Eötvös Loránd University, Rákóczi út 7, Budapest, 1088 Hungary; 7 Health Policy Research Group, University of Oregon, 1209 University of Oregon, Eugene, OR 97403-1209 USA

**Keywords:** PAM-13, Patient activation, eHEALS, Health literacy, Lifestyle-related risks, Online survey, Psychometric validation, I10, I10

## Abstract

**Background:**

Patient activation comprises the skills, knowledge and motivation necessary for patients’ effective contribution to their care. We adapted and validated the 13-item Patient Activation Measure (PAM-13) in the ≥ 40 years old Hungarian general population.

**Methods:**

A cross-sectional web survey was conducted among 900 respondents selected from an online panel via quota sampling. After 10 days, the survey was repeated on 100 respondents. The distribution, internal consistency, test–retest reliability, factor structure, convergent, discriminant and known-groups validity of PAM-13 were assessed according to the COSMIN guidelines.

**Results:**

The sample comprised 779 respondents. Mean (± SD) age was 60.4 ± 10.6 years, 54% were female and 67% had chronic illness. Mean (± SD) PAM-13 score was 60.6 ± 10.0. We found good internal consistency (Cronbach alpha: 0.77), moderate test–retest reliability (ICC: 0.62; *n* = 75), a single-factor structure and good content validity: PAM-13 showed moderate correlation with the eHealth Literacy Scale (*r* = 0.40), and no correlation with age (*r* = 0.02), education (*r* = 0.04) or income (*ρ* = 0.04). Higher PAM-13 scores were associated with fewer lifestyle risks (*p* < 0.001), more frequent health information seeking (*p* < 0.001), participation in patient education (*p* = 0.018) and various online health-related behaviours. When controlling for health literacy, sociodemographic factors and health status, the association of higher PAM-13 scores with overall fewer lifestyle risks, normal body mass index, physical activity and adequate diet remained significant. Similar properties were observed in the subgroup of participants with chronic morbidity, but not in the age group 65+.

**Conclusion:**

PAM-13 demonstrated good validity in the general population. Its properties in clinical populations and the elderly as well as responsiveness to interventions warrant further research.

**Supplementary Information:**

The online version contains supplementary material available at 10.1007/s10198-022-01434-0.

## Background

In our days more and more people live with chronic conditions, which will account for nearly three quarters of global mortality in 2020 [1]. The quest for sustainable financing of healthcare systems needs to embrace efficient approaches in the management of chronic conditions [2]. Over the last decades, patient centredness has gained considerable attention in medicine, which, by putting patients’ values and preferences in the forefront of medical decision-making, aims for their more efficient involvement as partners in the health production process [3, 4]. The monitoring of patient-reported outcomes (PROs) is becoming a strategic priority for healthcare systems [5]. Self-reported health, disability and chronic morbidity (measured by the Minimum European Health Module, MEHM) are now routinely collected in Europe-wide harmonised statistical surveys [6, 7]. The active involvement of patients is particularly important in the reduction of modifiable lifestyle-related risks, which contribute to a considerable share of excess mortality from chronic conditions [8]. Large-scale policy measures, such as changes of income or education, that can influence important individual determinants of health and healthy behaviours potentially span in time over generations. Therefore, personally acquired potentials, which can develop over later courses of life, such as knowledge, skills, positive emotions and engagement are of particular importance from both public health and health economic perspective [9, 10]. A number of theories, such as internal locus of control [11], self-efficacy [12], self-management [13] or the transtheoretical model of change [14], have addressed the drivers of change in health behaviours, and a number care delivery models, such as the Chronic Care Model, promoted systematic improvements involving patient centredness, support for self-management, evidence-based proactive interventions, integrated team care and supportive information technology solutions [15]. Digital health interventions have been shown to be effective in promoting healthy behaviours through patient education or supporting behaviour change, and upon the demonstration of adequate supportive evidence, authorities are now considering their adoption among publicly financed health technologies [16, 17].

The knowledge component of patients’ potential for their effective contribution to health production is usually conceptualised as health literacy: “the degree to which individuals have the ability to find, understand, and use information and services to inform health-related decisions and actions for themselves and others” [18]. Electronic health literacy also involves the skills to appraise information from electronic sources [19]. Health literacy can be assessed from objective performance (e.g. Newest Vital Sign, NVS) [20] self-reported skills (e.g. Electronic Health Literacy Scale, eHEALS) [19] or indirectly from self-reported behaviours [21]. Although the association between health literacy and mortality benefits has been shown, the evidence concerning its association with better health outcomes or healthy behaviours in chronic conditions is mixed [22, 23].

The concept of patient activation encompasses a broad range of elements beyond health literacy, that enable patients to become effective and informed managers of their health [24]. Patient activation and health literacy are moderately correlated and contribute differently to outcomes [25]. The Patient Activation Measure (PAM) has been developed to serve as a reliable tool that can measure the skills, knowledge and motivation of patients that are necessary for their effective contribution to their own care, and eventually predict better outcomes [24]. While health literacy is associated with better understanding and use of information for making informed choices, patient activation is more strongly associated with healthy behaviours and outcomes [25]. Since its development, PAM has become an officially adopted patient-reported outcome measure (PROM) by the National Institute of Health of the US and the National Health Service of the UK. Its validated versions have been available in over 20 countries and it has been applied in over 500 studies worldwide [26]. It has been demonstrated that higher PAM values are associated with better health outcomes [27], fewer lifestyle-related risks [28], better adherence to therapy [29] and lower use of healthcare resources [28, 30]. Furthermore, it has been shown that patient activation can be improved via digital health interventions [31, 32] as well as offline patient support programmes [33].

In accordance with national policies aiming to reduce lifestyle-related excess mortality as well as the advancement of digital health [34], our aim was to adapt and validate the 13-item PAM-13 tool in the Hungarian general population to serve as a widely tested and internationally recognised instrument in the development or monitoring of evidence-based health promotion interventions. In addition to assessing its psychometric properties, we aimed to establish the validity of PAM-13 by demonstrating its association with self-reported skills and behaviours that are expected to signal the effective contribution of individuals to their own health or health care. Specifically, we tested whether higher scores of PAM-13 are positively associated with health literacy, health preventive behaviours, healthier lifestyle as well as engagement with health information seeking, patient education and online health behaviours, such as health-related communication, health information seeking or participation in disease prevention or disease management programmes.

## Methods

### Study design and participants

In April 2020 we conducted an online survey recruiting 900 respondents aged ≥ 40 years from a large online panel via quota-based sampling with strata set according to the 2011 population census [35]. As multiple PAM-13 items inquire experiences related to medical care or disease management, we omitted younger adults from the sample to ensure sufficiently high proportion of valid responses (see below). Our sample was representative of the Hungarian population in terms of age groups, gender, education, geographic region and type of settlement. After 10 days, 100 respondents were randomly selected for repeated administration of the entire survey. We considered 10 days lag sufficient to prevent recall while capturing a stable PAM-13 status [36–38]. Ethical approval was granted by the National Medical Research Council (TUKEB, ID: 49702-3/2019/EKU) and a research license was obtained for PAM-13. Recruitment and data collection were performed by an online research company; all participants provided consent prior completing the survey.

### Measurement tools and survey items

#### PAM-13 and development of the Hungarian language version

PAM assesses one’s knowledge, skill and confidence for self-management. The original version consisted of 22 items. The short version PAM-13 instrument consists of 13 items, scored on a 4-point Likert scale and a 5^th^ ‘not applicable’ option (Online Resource 1). For a valid PAM-13 score, up to 3 ‘not applicable’ responses were allowed. Using a proprietary scoring algorithm based on Rasch analysis, PAM-13 is scored on a scale of 0–100, where lower values suggest less likelihood that patients engage in effective self-management. Based on their PAM-13 scores, patients can be grouped into four PAM-13 levels. At level 1, patients may not understand the need to take active role in their health. At level 2, their confidence or skill is probably too low to take action. At level 3, patients are beginning to take action, and at level 4, they may endure in self-management even in difficult times [24, 39, 40]. In this analysis, we use both the 0–100 score and the 4 levels of activation.

The Hungarian language version of the PAM-13 questionnaire was produced in accordance with the WHO guidelines for the translation and adaptation of instruments [41]. Forward translation was performed by two independent experts and the back translation was carried out by two bilingual translators and elaborated by two researchers (DÁ and ZZ) against the original version of the instrument. The draft instrument was piloted along with cognitive debriefing on 10 respondents, including both males (*N* = 3) and females (*N* = 7), from different age groups (mean ± SD age 46.8 ± 16.9 years). The literal translations were overridden at several questions with natural phrases that were considered to be acceptable for the broadest audience, yet conceptually equivalent with the original questionnaire. The pre-final version was consulted with the developers of the PAM instrument. The 4-level Likert scale response options of the original instrument (“Disagree strongly”, “Disagree”, “Agree”, “Agree strongly”) were slightly modified to mark more precisely the scale degrees in a Hungarian context (“Completely disagree”, “Rather disagree”, “Rather agree”, “Completely agree”). The Hungarian PAM-13 is attached in Online Resource 1.

#### eHEALS

The eHealth Literacy Scale (eHEALS) measures self-reported eHealth literacy using eight 5-point Likert scale items. The eHEALS score (range 8–40) is calculated by summing individual item scores, with higher values indicating greater skill levels. The Hungarian eHEALS instrument has been validated in the general population via an online survey [19, 42]. Since eHEALS showed weak correlation with objective performance tests [43, 44], we also measured performed health literacy in our survey.

#### Newest vital sign

The newest vital sign (NVS) is a frequently used screening instrument for performed health literacy. Respondents need to answer six questions by interpreting the information from an ice cream nutritional label and performing simple arithmetic tasks. For some questions correct answers can be formulated in several ways. The number of correctly answered items is counted. A score of 0–1 indicates limited, 2–3 indicated probably limited and 4–6 indicates adequate health literacy [20]. We adapted the Hungarian NVS for online administration [45]. Instead of offering multiple-choice options in the online adaptation [46], we specified the measurement unit for answers in the questions and evaluated the accuracy of free-text answers.

#### Minimum European Health Module

We also inquired respondents’ health by the Minimum European Health Module (MEHM). The MEHM consists of three questions. Self-perceived health evaluates current health on a 5-point Likert scale (“Very good”; “Good”; “Fair”; “Bad”; “Very Bad”). The Global Activity Limitation Indicator (GALI) asks limitations in activities over the past 6 months due to a health problem (“Not limited”, “Limited but not severely”, “Severely limited”). A final item (chronic morbidity) inquires the presence of long-standing health problems [6].

#### Health-related information seeking and online behaviours

Following the indirect measurement strategy of the European citizens’ digital literacy survey [21], we included seven items to assess the frequency of various health-related information seeking and online behaviours over the past 12 months. Item 1 inquired about health information seeking in general and item 2 about participation at patient education programmes. Items 3–7 inquired about health-related use of the internet or mobile devices in different functional domains, motivated by the classification of the evidence standards framework of digital health interventions proposed by The National Institute for Health and Care Excellence (NICE) [17]. Item 3 inquired general online health-related administration, item 4 about online health-related information seeking, item 5 about online health-related communication with healthcare professionals, online helpers or peer patients, item 6 about online health prevention activities and item 7 about online disease management activities. All items were scored on a 6-point Likert scale (“Never”, “Few times past year”, “Bimonthly”, “Monthly”, “Several times per month”, “At least once a week”). For hypothesis testing, we dichotomised each item to high and low activity at the median category.

#### Demographic variables

We recorded basic demographic variables age, gender, education, type of settlement, region and the place of residence based on postcode. Net monthly household income was queried in 11 range categories, and per-capita household income was calculated by dividing the category mid-range values by the number of household members, without adjustment for the number of children. The mid-range value of the upper open category was calculated by fitting the Pareto curve as proposed by Parker and Fenwick [47]. Local currency values were transformed to Euros using the average exchange rate for the period of Apr 1, 2019 Apr 1, 2020 (€1 = 330.7 HUF) [48].

#### Lifestyle risk factors

We recorded the most common modifiable risk factors for all-cause mortality and chronic conditions, such as BMI, smoking, alcohol intake, dietary habits, physical activity and sedentary behaviour [49–53]. Lifestyle risks were inquired via single-question self-reported items. In order to represent similar “severity levels”, the following cut-off values were chosen that represent approximately 1.4-fold or greater relative risk increase for overall mortality: BMI < 18.5 or BMI ≥ 30 [54], current smoking [55, 56], sedentary behaviour ≥ 8 h per day with < 150 min exercise per week or no exercise at all [57], no fruit and/or vegetable intake [58] and binge drinking ≥ 1 day per week [59]. Binge drinking was defined as > 5 drinks/occasion for men and > 4 drinks/occasion for women [59, 60]. We also generated a lifestyle risk index by adding the number of lifestyle risk factors for each patient. Based on their lifestyle risk index, respondents were assigned to risk groups using stringent (no lifestyle risk vs any lifestyle risks) and relaxed (0–1 vs 2–4 lifestyle risk factors) criteria.

#### Preventive behaviours

We considered as preventive behaviours the participation at screenings and vaccination programmes which are officially recommended in Hungary [61, 62]. According to this, for females, we counted the participation at cervical cancer screening between 25 and 65 years of age, breast cancer screening between 45 and 65 years of age, colorectal screening between 50 and 70 years of age, blood pressure, blood glucose and cholesterol levels measured within a year, flu vaccination at 60+ years of age and bacterial pneumonia vaccination at 50+ years of age. For males, we counted the participation at prostate and colorectal cancer screening between 50 and 70 years of age, blood pressure, blood glucose and cholesterol levels measured within a year, flu vaccination at 60+ years of age and bacterial pneumonia vaccination at 50+ years of age. To make respondents with different gender and age comparable, we calculated the preventive behaviour score as the proportion of performed preventive behaviours compared to the maximum of preventive behaviours prescribed for a given age and gender. For example, having only blood pressure measured within a year would represent a preventive behaviour score of 0.33 for a 40-year-old man (with only blood pressure, glucose and cholesterol check recommended), while it would be a score of 0.125 for a 60-year-old woman, (for whom cervix, breast and colorectal cancer screenings, blood pressure, glucose and cholesterol tests as well as flu and bacterial pneumonia vaccinations are recommended). We also grouped respondents based on their preventive behaviour score (< 50%, ≥ 50%).

### Excluded respondents

Before data analysis, we checked the dataset for outliers and based on group consensus, set implausible values to missing or deleted entire records in case of potentially unreliable answer patterns. We deleted the data point if sedentary time was reported > 18 h/day. We deleted cases if the frequency of online health information seeking was reported over two categories greater than general health information seeking, response times shorter than 4 s per item for the survey instruments (PAM-13, eHEALS) or shorter than 1 min for the NVS instrument [63]. One respondent was excluded due to unlikely body parameters (height 111 cm, weight 200 kg, BMI = 162), and based on the PAM license owner’s recommendation, we dropped individuals with a PAM-13 score of 0 and 100 (respondents who answer all strongly agree or all strongly disagree are likely not paying attention and responding in a valid way) as well as ones with not applicable answers in more than 3 PAM-13 items [64].

### Statistical methods

We followed the applicable COSMIN guidelines for patient-reported outcome measurement instruments when planning the methods of our study [65–67]. Missing data, descriptive statistics and distributional properties were assessed for all variables. The distribution of PAM-13 scores was assessed via inspection of the histogram and quantile plot, and normality was tested via the Shapiro–Wilk test. Floor and ceiling effects were assessed against the threshold of 15% [68]. We tabulated respondents based on their PAM-13 levels. All analyses were performed on unweighted data.

#### Reliability

We evaluated internal consistency via computing Cronbach’s alpha. Test–retest reliability for PAM-13 scores was assessed by intra-class correlation coefficient of agreement using a two-way random effects model (ICC_agreement_ or ICC(A,1)) [69]. For categorical PAM-13 levels, we calculated weighted kappa using quadratic weights to progressively penalise greater differences between categories [69]. Measurement error (standard error of measurement, SEM) was calculated using the formula $\mathrm{SEM}=\sigma \times \sqrt{1-{\mathrm{ICC}}_{\mathrm{agreement}}}$, where $\sigma$ is the pooled standard deviation of the sample from first and repeat administrations. The smallest detectable change (SDC, the smallest change that can be detected within a single person with p < 0.05 taking measurement error into consideration) was calculated via the following formula: $\mathrm{SDC}=1.96\times \sqrt{2\times \mathrm{SEM}}$ [38]. We considered the following thresholds for good measurement properties: ≥ 0.7 for ICC_agreement_ and weighted kappa, and the range of 0.7–0.95 for Cronbach’s alpha [38].

#### Validity

Content validity was assessed during the translation process; no further quantitative measurements were performed. Construct validity was assessed via confirmatory factor analysis using robust structural equitation modelling via the R package lavaan [70], assuming a single underlying factor. We checked the Kaiser–Meyer–Olkin (KMO) statistic for the adequacy of sampling [71] and Bartlett test for sphericity to check the adequacy of our data for factor analysis. Model fit was assessed by the RMSEA, the Tucker–Lewis index (TLI) and the comparative fit index (CFI), using cut-off values of ≤ 0.05, 0.9 and 0.9 for good fit, respectively. Convergent validity was assessed by the correlation between PAM-13 scores as well as PAM-13 levels and eHEALS scores, based on the assumption that both instruments measure advanced knowledge and are conceptually related to self-efficacy [19, 24]. We expected significant positive relationship between the two measures. Discriminant validity was tested by the expectation of weak or non-significant correlation between PAM-13 scores as well as PAM-13 levels and age, education and income, based on the assumption that PAM measures qualities that cannot be explained by socioeconomic status. We applied Pearson correlation between continuous measures, and polyserial correlation when categorical measures were involved.

When testing known-groups validity, our hypothesis was the following: patients practicing more preventive behaviours (preventive behaviour score ≥ 50%), having fewer lifestyle risk factors according to the stringent (lifestyle risk index = 0) or relaxed (lifestyle risk index ≤ 1) criteria, those, who were more active in health information seeking, patient education, health-related communication, online/mobile health information seeking, disease prevention or disease management activities had higher mean PAM-13 scores [24, 72]. We defined higher activity as having at least median scores on each item, or any activity over the past year, if majority of respondents did not engage in the respective online activity. The hypotheses were tested using one-sided Welch’s *t* test. We also explored the same hypotheses in subgroups of patients with or without chronic disease, male or female respondents, respondents ≥ 65 years of age or younger, respondents in the lowest income group or higher and respondents with adequate (NVS ≥ 4) or lower health literacy scores. We deleted missing values pairwise for all statistical analyses. No missing values were imputed.

#### Rasch analysis

Insignia Health (copyright owner of PAM-13) provided the authors with PAM-13 scores based on the Rasch model [39, 73] as well as values of infit and outfit indices for each participant in both administrations of the survey. These indices measure the goodness-of-fit of the model to the observed response patterns. Infit is more sensitive to deviations on items aimed at the true activation levels of the respondents, whereas outfit is more sensitive to deviations on items aimed far from their true activation levels. Several thresholds for person infit and outfit indices exist in the literature, and according to one popular system [74], values below 0.3 imply a lack of expected variability in answers, values between 0.3 and 3.0 indicate good to high-quality data and values greater than 3 indicate unusual response patterns and thus poor-quality data [75, 76].

#### Regression analysis

We tested the association of PAM-13 score with the preventive behaviour score, lifestyle risk index, individual lifestyle-related risk factors (BMI, smoking, alcohol, physical activity and diet), health information seeking, participation at patient education and online health-related behaviours, when controlled for eHealth literacy (eHEALS), health literacy (NVS), age, gender, education, income, type of settlement and the health status of the respondent (MEHM items). We also replaced PAM-13 scores with PAM-13 levels. For continuous variables we applied OLS regression. Heteroskedasticity and model specification was tested via the Breusch–Pagan [77] and Ramsey RESET tests [78], respectively. In case of heteroskedasticity, we applied robust regression. Binary risk factors were analysed via logistic regression and the ordinal health information seeking and online behaviours via ordered logit models. For logistic and ordered logit models, goodness-of-fit was assessed via the binary and multinomial versions of the Hosmer–Lemeshow test [79, 80].

## Results

### Descriptive statistics

From the 900 survey respondents due to PAM-13-related quality issues, we excluded 92 respondents (10.2%) and 29 respondents (3.2%) for other reasons detailed above. Altogether 779 (86.6%) individuals were included in our sample. In the repeat survey (*n* = 100), we excluded 11 respondents (11.0%), who had PAM-13-related quality issues, 4 (4.0%) due to other reasons and 10 respondents, who were excluded in the first administration. The retest sample comprised 75 respondents with had matching test–retest scores.

In the sample, mean age was 60.4 (SD = 10.6) years, 54.0% were female and 66.5% reported to have chronic disease. Highly educated, affluent urban respondents were over-represented compared to the 40+ year-old general population (Table 1). The demographic characteristics of survey respondents and reasons for exclusion are summarised in Online Resource 2.

**Table 1:** Sociodemographic characteristics and health status

	Sample		Retest sample		General population 2011^a^
	*n*	%	%	%	%
Total	779	–	75	–	–
Age group					
40–49	143	18	14	15	26
50–59	177	23	14	21	28
60–69	306	39	11	37	23
70+	153	20	9	27	23
Gender					
Male	358	46	44	49	44
Female	421	54	56	51	56
Education					
Primary	203	26	62	45	35
Secondary	288	37	28	33	49
Tertiary	288	37	10	21	16
Region					
Central Hungary	276	35	22	29	29
Central Transdanubia	83	11	10	13	11
Western Transdanubia	69	9	7	9	10
Southern Transdanubia	89	11	9	12	10
Northern Hungary	79	10	5	7	12
Northern Great Plain	85	11	16	21	15
Southern Great Plain	98	13	6	8	13
Type of settlement					
Capital	181	23	17	24	17
Town	447	57	52	48	52
Village	151	19	31	28	31
Income					
1st quintile	75	11	20	12	
2nd quintile	106	16	20	20	
3rd quintile	74	11	20	13	
4th quintile	122	18	20	17	
5th quintile	291	44	20	38	
Missing	111	14	6	8	
Self-rated health					
Very good	39	5	2	3	
Good	267	34	25	33	
Fair	397	51	39	52	
Bad	66	8	8	11	
Very bad	10	1	1	1	
Missing	0	0	0	0	
Chronic morbidity					
No	253	33	20	27	
Yes	503	67	54	73	
Missing	23	3	1	1	
GALI					
Not limited	496	64	40	53	
Limited but not severely	243	31	32	43	
Severely limited	37	5	3	4	
Missing	3	0	0	0	

^a^Population census 2011[35]

Mean (± SD) PAM-13, eHEALS and NVS scores were 60.6 (± 10.0), 28.7 (± 5.1) and 3.9 (± 1.7), respectively. The number (%) of patients with PAM-13 levels 1, 2, 3 and 4 were 54 (6.9%), 169 (21.7%), 471 (60.5%) and 85 (10.9%), respectively. Health literacy measured by the NVS was adequate (≥ 4) for 491 (63.0%), possibly limited (2–3) for 197 (25.3%) and probably limited (≤ 1) for 91 (11.7%). Mean (± SD) lifestyle risk index was 1.2 (± 1.0). Out of 779 respondents 0, 1, 2 and ≥ 3 lifestyle risk factors were reported by 210 (27.0%), 275 (35.3%), 207 (26.6%) and 87 (11.2%), respectively. Mean (± SD) preventive behaviour score was 0.45 (± 0.25). According to the cut-off values for hypothesis testing, 466 (59.8%) respondents searched health-related information at least monthly, 171 (22.0%) participated in patient education over the past year, 443 (56.9%) performed health-related administration over the internet, 421 (54.0%) sought health-related information at least bimonthly, 196 (25.2%) communicated online over past year about health with healthcare professionals, helpers or peers, 280 (35.9%) engaged in online health prevention activities and 347 (44.5%) participated in online disease management activities over the past year (Online Resource 3).

### Classic test theory methods

The distribution plots of PAM-13 are displayed in Online Resource 4. We summarised the psychometric properties of PAM-13 along with the applied methods, target values and results in Table 2. Altogether, PAM-13 scores fit rather log-normal than normal distribution with no floor or ceiling effect. Internal consistency was adequate, while test–retest reliability was moderate. The single-factor structure was confirmed, convergent validity (correlation with eHEALS scores) and discriminant validity hypotheses (low/no correlation with age, education and income) were supported. Known-groups validity tests were significant in 7/9 (77.8%) hypotheses.

**Table 2:** Summary of the results of classical test theory methods

Category	Property	Method	Target	Result	*p* value/[95%CI]	Comment
General	Distribution	Skewness	0.00	0.36	< 0.001	Positive skew
		Kurtosis	3.00	3.24	0.165	Normal kurtosis
		Shapiro–Wilk test for normal distribution	*p* ≥ 0.05	–	< 0.001	Deviation from normality
		Shapiro–Wilk test for log-normal distribution	*p* ≥ 0.05	–	0.811	Log-normal distribution
		Floor effect	< 15%	0.13%	[0.0–0.7%]	No floor effect
		Ceiling effect	< 15%	0. 25%	[0.0–0.9%]	No ceiling effect
Reliability	Internal consistency	Cronbach alpha	0.7–0.95	0.77	[0.74–0.79]	Adequate
	Test–retest reliability	ICC_agreement_^a^	> 0.7	0.62	[0.46–0.74]	Moderate
		Standard error of measurement	–	6.5	[5.4–7.8]	–
		Smallest detectable change	–	7.1	[6.4–7.7]	–
		Weighted kappa^b^	> 0.7	0.46	[0.26–0.65]	Moderate
Validity	Structural validity (CFA^c^)	Sample: KMO^d^	0.5	0.84	–	Adequate
		Sample: Bartlett test	*p* < 0.05	–	< 0.0001	
		Single factor: RMSEA^e^	< 0.05	0.049	[0.041–0.057]	Good fit
		Single factor: CFI^f^	> 0.90	0.947	–	
		Single factor: TLI^g^	> 0.90	0.937	–	
	Convergent validity	PAM-13—eHEALS^h^ Pearson correlation	*r* > 0.3^i^	*r* = 0.40	< 0.001	Supported
		PAM-13 levels—eHEALS Polyserial correlation^b^	*ρ* > 0.3^i^	*ρ* = 0.39	< 0.001	Supported
	Discriminant validity	PAM-13—age Pearson correlation	*r* < 0.3^j^	*r* = 0.02	0.524	Supported
		PAM-13—education polyserial correlation	*ρ* < 0.3^j^	*ρ* = 0.04	0.273	Supported
		PAM-13—income quintiles polyserial correlation	*ρ* < 0.3^j^	*ρ* = 0.04	0.321	Supported
	Known-groups validity (PAM-13 score difference)	PBS^k^ ≥ 50% vs PBS < 50%	Δ > 0.0^i^	Δ = 0.91	0.102	Not supported
		Lifestyle risk index:0 vs ≥ 1	Δ > 0.0^i^	Δ = 3.87	< 0.001	Supported
		Lifestyle risk index ≤ 1 vs ≥ 2	Δ > 0.0^i^	Δ = 4.47	< 0.001	Supported
		Health information seeking at least monthly vs less	Δ > 0.0^i^	Δ = 2.41	< 0.001	Supported
		Patient education over past year vs none	Δ > 0.0^i^	Δ = 1.88	0.018	Supported
		Online health information seeking at least bimonthly or less	Δ > 0.0^i^	Δ = 0.91	0.104	Not supported
		Online health-related communication past year vs none	Δ > 0.0^i^	Δ = 1.61	0.025	Supported
		Online health-prevention over past year vs none	Δ > 0.0^i^	Δ = 1.60	0.015	Supported
		Online disease management over past year vs none	Δ > 0.0^i^	Δ = 1.46	0.044	Supported

^a^ICC: intra-class coefficient

^b^Results refer to PAM-13 levels (all other results: PAM scores)

^c^CFA: confirmatory factor analysis

^d^KMO: Kaiser–Meyer–Olkin statistic

^e^RMSEA: root mean squared error of approximation

^f^CFI: comparative fit index

^g^TLI: Tucker–Lewis Index

^h^eHEALS: eHealth Literacy Scale

^i^*p* < 0.05, significant

^j^*p* ≥ 0.05, not significant

^k^PBS: preventive behaviour score

The results of known-groups hypothesis tests in various subgroups are summarised in Online Resource 5. Generally fewer than 7 hypotheses were supported in the subgroups than in the entire sample, except for participants with chronic morbidity (*n* = 503, 7/9 hypothesis supported) and 40–65 years old (*n* = 483, 8/9 hypothesis supported). None of the hypotheses were supported in the > 65 age group (*n* = 296). Respondents with higher PAM-13 levels had fewer lifestyle risk factors in 9/10 subgroups and sought more often health information in 7/10 subgroups. The smallest detectable change (SDC) was 7.1 points.

### Rasch analysis

Table 3 summarises the data quality of the Rasch model based on the person infit and outfit indices. The results indicate that the PAM-13 scores used in this paper are based on high-quality, consistent data.

**Table 3:** Summary of Rasch data quality indices

	First administration		Second administration	
	Good to high	Poor	Good to high	Poor
Infit	732 (94.0%)	47 (6.0%)	72 (96.4%)	3 (3.6%)
Outfit	728 (93.5%)	51 (6.5%)	72 (96.4%)	3 (3.6%)

### Regression analysis

When controlled for eHealth literacy, health literacy, sociodemographic variables and respondents’ health status, the association of PAM-13 scores with lifestyle-related risks remained significant. In particular, higher PAM-13 scores were associated with fewer lifestyle-related risks overall, lower probability of risky BMI (< 18.5 or > 30), physical inactivity or diet low in fruits and/or vegetables. However, smoking and binge drinking episodes were not associated with PAM-13. Furthermore, although health information seeking, participation at patient education programmes and several online health-related behaviours were associated with PAM in bivariate hypothesis tests, in multiple regression models these factors were associated with eHEALS or NVS but not with PAM-13 scores. Higher preventive behaviour scores were associated with higher education levels, but neither with patient activation (PAM-13 score or level) nor with health literacy (eHEALS or NVS). The regression table is shown in Online Resource 6.

**Fig. 1:**
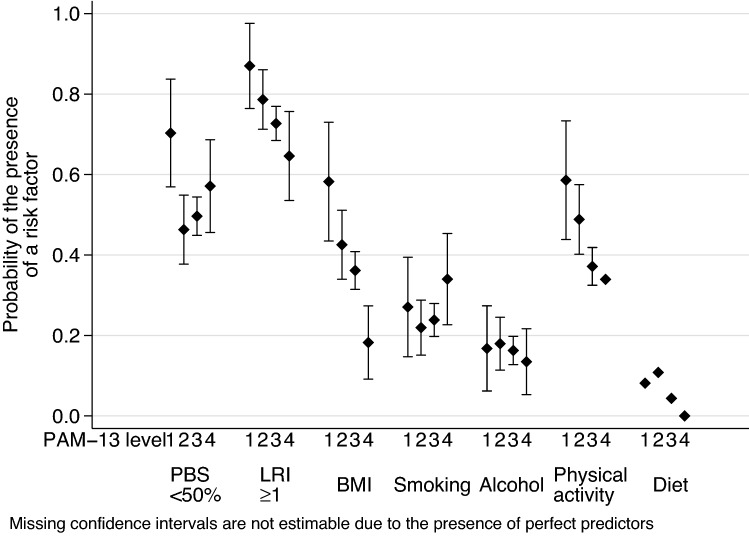
Adjusted probability of lifestyle-related risks at various PAM-13 levels in the entire sample. *PBS* preventive behaviour score, *LRI* lifestyle risk index, *BMI* body mass index

**Fig. 2:**
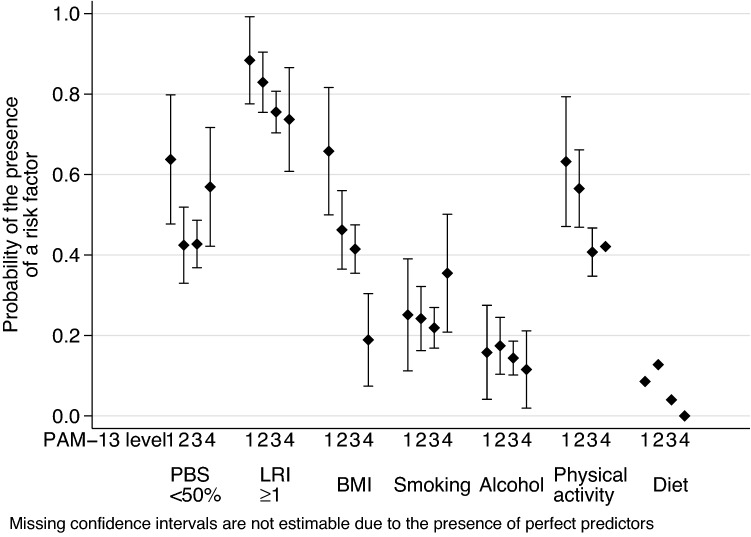
Adjusted probability of lifestyle-related risks at various PAM-13 levels in the subgroup with chronic morbidity. *PBS* preventive behaviour score, *LRI* lifestyle risk index, *BMI* body mass index

**Fig. 3:**
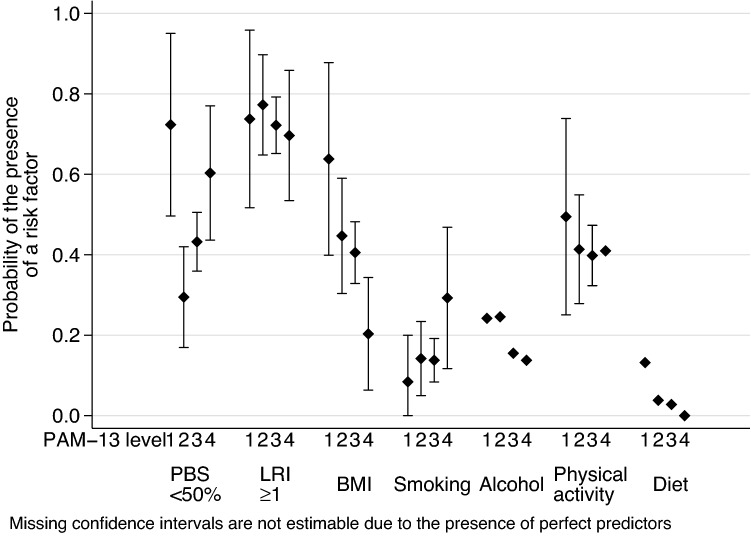
Adjusted probability of lifestyle-related risks at various PAM-13 levels in the 65+ subgroup. *PBS* preventive behaviour score, *LRI* lifestyle risk index, *BMI* body mass index

PAM-13 levels showed similar pattern to PAM scores (Online Resource 6). After controlling for the regression predictor variables, the adjusted probabilities of various lifestyle-related risks at different PAM-13 levels are depicted in Fig. 1 for the entire sample, in Fig. 2 for the subgroup with chronic morbidity and in Fig. 3 for 65+ year-old patients. While the pattern of participants with chronic morbidity was similar to the entire sample, neither the overall number of risk factors nor the level of physical activity was associated with higher PAM-13 levels in the elderly (65+) subgroup (Online Resources 8, 9 and 10).

## Discussion

In this cross-sectional online survey among the 40+ year-old Hungarian general population, we demonstrated the validity and tested the psychometric properties of the Hungarian version of the PAM-13 instrument. Following the COSMIN guidelines, we found good internal consistency, moderate test–retest reliability, a single-factor structure and good content validity consistent with majority of the predefined hypotheses. In particular, higher PAM-13 scores of PAM-13 levels were associated with fewer lifestyle-related risks even when controlled for eHealth literacy, health literacy, socioeconomic characteristics and the health status of respondents. Higher PAM-13 scores were especially associated with greater probability of having normal BMI, being physically active and having a diet, including vegetables and fruits. However, smoking and risky alcohol intake were not associated with PAM-13 scores.

The results of bivariate hypothesis tests were consistent with the entire sample in the subgroup of respondents with self-reported chronic morbidity and in the 40–65 years old age group. However, in the 65+ population higher PAM-13 levels were not associated with either fewer lifestyle risk factors or more health information seeking, participation at patient education or online health-related activities. Albeit not significant, the pattern of regression coefficients of lifestyle risk factors, BMI, physical activity and diet on PAM-13 score in the 65+ age group were similar to those in the general population (Online Resource 10). Therefore, the validity of PAM-13 in the elderly population should be further studied with larger population samples and confirmed by testing alternative hypotheses more relevant to this population, such as disease management, medication adherence [29] or costs of care [40].

Our results are consistent with other validation studies. Comparisons are more relevant with surveys that were conducted among the general population; however, the differences between the demographic characteristics of the samples should be considered. The PAM instruments were developed on a sample of 45+ year-old individuals from the US general population, with 34% over age of 65 [24, 39]. Fowles et al. assessed the validity of PAM-13 in a younger age group: US employees with mean age 45 of years [81]. PAM-13 was also validated in the general population of Israel (age 65+: 26.7%) [82] and the Netherlands (age 65+: 29.3%) [83]. The average PAM-13 score varied between 60.2 and 70.7 across the four studies, our result was closest to the original development study (60.6 vs. 60.2) [39]. Cronbach alpha varied between 0.77 and 0.9 [24, 39, 82, 83]. Associations between PAM-13 and demographic characteristics (age, gender, educational level) varied across the four studies; however, positive association with health status (assessed by different measurement tools) was rather consistent [24, 39, 82, 83].

We tested the association between patient activation and a broad set of variables concerning participants’ health-related risk factors, information seeking and health management behaviour. Similarly to our findings, Hibbard et al. reported that the preventive, consumeristic and the disease-specific self-management behaviours were strongly associated with PAM-13 scores [39]. Fowles et al. found strong positive association between PAM-13 and personal healthy behaviours and health information seeking [81]. While online health information seeking was not associated with PAM-13 scores in our entire sample, in younger individuals and ones without chronic disease the association was positive. Health information seeking in general was not associated with PAM-13 scores in the elderly. As shown by our regression analyses and suggested by previous research, patient activation is rather associated with health behaviours, while information seeking is rather associated with health literacy [25].

The aim of PAM-13 is to measure skills, knowledge and motivation necessary for patients’ effective contribution to their own care, and eventually predict better outcomes [24, 39]. In our study, the items of MEHM were used to control for the health status of respondents. However, assuming diverse health-related behaviours and health states in the general population, the association between PAM-13 and health status was not tested in this study.

PAM-13 validation studies were conducted also in a series of specific patient populations, including diabetes [37, 84, 85], metabolic syndrome [86], multiple sclerosis [87], neurological disorders [88, 89], hypertension [37, 85], cardiac conditions [90], schizophrenia [91], mental disorders [92], osteoarthritis [93–95], rheumatic diseases [37, 96], HIV [97] or not specified chronic diseases [98–100], and clinical settings in different countries [101–105]. In some of these studies, convergent validity was assessed using generic and disease-specific measures of health-related attitudes or behaviours, such as self-efficacy [87, 88, 96, 101], self-esteem [98], lifestyle [89] or health literacy [103, 106]. For the assessment of convergent validity, we chose the eHEALS scale, a widely used measure of self-reported eHealth literacy that is strongly rooted in self-efficacy theory [19, 42]. While PAM-13 and eHEALS showed moderate positive correlation, the multiple regression analyses revealed that the two instruments are associated with different facets of health-related behaviours. While higher PAM-13 scores predicted healthier lifestyles, higher eHEALS scores were associated with more frequent health information seeking and online health-related behaviours.

The strength of our study is that it met the most meticulous methodological standards outlined by the COSMIN best practices guide [65]: all phases of our approach, including descriptions of the research aim, the PROM and the target population, the design requirements, the analysis of structural validity, internal consistency, measurement invariance, measurement error and reliability, criterion validity, construct validity, known-groups validity and the construct approach (hypothesis testing), fell into the highest quality category defined by these guidelines.

We note that 92 out of 900 respondents (10.2%) were excluded from the survey due to PAM-13-related quality issues, following the developers’ recommendations [64]. While more than three “not applicable” responses (*N* = 51, 5.6%) may indicate that a respondent lacks relevant health-related experiences, “straightlining” patterns (e.g. all “disagree strongly” or “agree strongly” on all items, *N* = 41, 4.6%) are potential indicators of meaningless responses, especially given the Guttman scale-like structure of PAM-13, which involves items with increasing difficulty levels to reflect the developmental model of patient activation [24, 64] Due to other potential indicators of low data quality, such as low response times or grossly inconsistent patterns, we excluded respondents sparingly (*N* = 29, 3.2%) to decrease measurement error, while avoiding interference with results [107].

The limitation of our study is that PAM-13 was administered in an online general population, with over-representation of highly educated, affluent urban respondents, which reflects the general sampling bias of online surveys [108]. However, patients from rural regions or lower socioeconomic status may be particularly prone to lifestyle risks and poor health status [109, 110]; therefore, the validity of the patient activation concept is of particular importance in these populations. Nevertheless, in subgroups with the lowest income and inadequate health literacy levels, higher PAM-13 scores were associated with fewer lifestyle risk factors and more frequent health information seeking in our study, which supports the validity of the PAM-13 instrument in these vulnerable subgroups. Furthermore, although the validity of PAM-13 was supported among respondents with self-reported chronic morbidity, further studies are required to assess the psychometric properties of PAM in clinical settings also including younger adults. While we assumed low applicability for many PAM-13 items in the general population below 40 years of age, clinical validation studies of PAM-13 should involve a wider spectrum of ages.

Furthermore, as a potentially modifiable enhancer of patients’ contribution to the health production process, the responsiveness of PAM-13 to interventions is of key interest [4, 111]. However, the responsiveness of PAM-13 is a poorly explored area. We found one validation study from Norway in which patients with chronic inflammatory arthritis attending a patient education programme were involved [96]. The low responsiveness was partly explained by the substantial ceiling effect in 10 of the 13 items. Our results suggest that the moderate test–retest reliability of PAM-13 necessitates sufficiently large sample sizes in adequately powered studies to demonstrate change. We encourage further research both among the general public and patient populations to get a better insight into responsiveness of PAM-13 to interventions.

We suppose that certain properties of PAM-13 may be context specific depending on the healthcare setting. In Hungary, the weak primary care system acts as a gatekeeper and offers limited choice of providers [112], while healthcare remains hospital centric and inefficient despite the low expenditure levels [113]. Therefore, while patient activation may be associated with more efficient use of healthcare resources [40], activated patients may also cost more by demanding more services. For example, Hungarian patients from low socioeconomic status may have poorer overall access to care [110]. High activation may contribute to lower utilisation of costly emergency care and increase the utilisation of routine care in this population. Altogether, while higher PAM-13 scores were associated with lower resource use in the US healthcare system [28, 30], the association of PAM-13 with costs of care warrants further research in the Hungarian context.

As a conclusion, the Hungarian version of the PAM-13 demonstrated good validity in the 40–65-year-old general population as well as among people with self-reported chronic morbidity. Further research is needed to establish the validity of PAM-13 in clinical populations as well as among the elderly and its responsiveness to interventions.

## Supplementary Information

Below is the link to the electronic supplementary material.

Supplementary file1 (PDF 1081 KB)

Supplementary file2 (PDF 1081 KB)

Supplementary file3 (PDF 1081 KB)

Supplementary file4 (PDF 1082 KB)

Supplementary file5 (PDF 1081 KB)

Supplementary file6 (PDF 1081 KB)

Supplementary file7 (PDF 1081 KB)

Supplementary file8 (PDF 1081 KB)

Supplementary file9 (PDF 1081 KB)

Supplementary file10 (PDF 1081 KB)

## Data Availability

Data are available upon reasonable request from the authors.

## Code availability

The code of statistical analyses is available upon reasonable request from the authors.
