# Supplementary material for: Validation of the PAM-13 instrument in the Hungarian general population 40 years old and above
Source: Eur J Health Econ. 2022 Jan 31;23(8):1341–55. doi: 10.1007/s10198-022-01434-0 (PMC9550701; doi:10.1007/s10198-022-01434-0)
Supplement: Supplementary file 1 — Supplementary file1 (PDF 1081 KB) [file 10198_2022_1434_MOESM1_ESM.pdf]

## Electronic Supplementary Material 1.

Zrubka Z, Vékás P, Németh P, Dobos Á, Hajdu O, Kovács L, Gulácsi L, Péntek M, *Validation of the PAM-13 instrument in the Hungarian general population*. European Journal of Health Economics 2021.

### 13 kérdésből álló Betegaktiváció Kérdőív® (PAM-13)

Az alábbiakban néhány olyan állítás szerepel, melyeket az emberek az egészségükkel kapcsolatosan szoktak mondani. Kérjük, jelölje be, hogy mennyire ért egyet - vagy nem ért egyet - azzal, hogy Önre jellemzőek ezek az állítások. Válaszai tükrözzék azt, amit önmagára nézve igaznak tart, és ne azt, amiről azt gondolja, hogy mások elvárnának Öntől.

Amelyik állítás nem alkalmazható Önre, annál jelölje meg a "nem jellemző" lehetőséget.

| | | | | | | |
|---|---|---|---|---|---|---|
| *1* | Összességében az én felelősségem, hogy vigyázzak a saját egészségemre | Egyáltalán nem értek egyet | Inkább nem értek egyet | Inkább egyetértek | Teljesen egyetértek | Nem jellemző |
| *2* | Az egészségemet leginkább az befolyásolja, hogy aktívan foglalkozom vele. | Egyáltalán nem értek egyet | Inkább nem értek egyet | Inkább egyetértek | Teljesen egyetértek | Nem jellemző |
| *3* | Biztos vagyok benne, hogy tudok segíteni az egészségemmel kapcsolatos problémák megelőzésében vagy csökkentésében. | Egyáltalán nem értek egyet | Inkább nem értek egyet | Inkább egyetértek | Teljesen egyetértek | Nem jellemző |
| *4* | Minden felírt gyógyszeremről tudom, hogy mi a hatása. | Egyáltalán nem értek egyet | Inkább nem értek egyet | Inkább egyetértek | Teljesen egyetértek | Nem jellemző |
| *5* | Biztos vagyok benne, hogy meg tudom állapítani, hogy egy egészségi problémával orvoshoz kell fordulnom, vagy magam is meg tudom azt oldani. | Egyáltalán nem értek egyet | Inkább nem értek egyet | Inkább egyetértek | Teljesen egyetértek | Nem jellemző |
| *6* | Biztos vagyok benne, hogy el tudom mondani az orvosnak az aggályaimat akkor is, ha ő nem kérdezi. | Egyáltalán nem értek egyet | Inkább nem értek egyet | Inkább egyetértek | Teljesen egyetértek | Nem jellemző |
| *7* | Biztos vagyok benne, hogy ha szükségem van rá, el tudom végezni az otthonra előírt kezeléseket. | Egyáltalán nem értek egyet | Inkább nem értek egyet | Inkább egyetértek | Teljesen egyetértek | Nem jellemző |
| *8* | Értem az egészségi problémáimat és azok lehetséges kiváltó okait. | Egyáltalán nem értek egyet | Inkább nem értek egyet | Inkább egyetértek | Teljesen egyetértek | Nem jellemző |
| *9* | Tudom, hogy egészségi problémáimra milyen kezelési lehetőségek állnak rendelkezésre. | Egyáltalán nem értek egyet | Inkább nem értek egyet | Inkább egyetértek | Teljesen egyetértek | Nem jellemző |
| *10* | Kitartó tudtam maradni, amikor életmódot változtattam (pl. helyes táplálkozás vagy testmozgás). | Egyáltalán nem értek egyet | Inkább nem értek egyet | Inkább egyetértek | Teljesen egyetértek | Nem jellemző |
| *11* | Tudom, hogyan előzzem meg az egészségi problémáimat. | Egyáltalán nem értek egyet | Inkább nem értek egyet | Inkább egyetértek | Teljesen egyetértek | Nem jellemző |
| *12* | Biztos vagyok benne, hogy találok megoldást, ha új egészségi problémáim merülnek fel. | Egyáltalán nem értek egyet | Inkább nem értek egyet | Inkább egyetértek | Teljesen egyetértek | Nem jellemző |
| *13* | Biztos vagyok benne, hogy ha életmódot változtatok (pl. helyes táplálkozás vagy testmozgás), akkor még stresszes időszakokban is kitartó tudok maradni. | Egyáltalán nem értek egyet | Inkább nem értek egyet | Inkább egyetértek | Teljesen egyetértek | Nem jellemző |

Insignia Health, LLC®. Patient Activation Measure © 2003-2020 University of Oregon.
Minden jog fenntartva.

**Bizalmas. Csak érvényes PAM® Licencmegállapodás mellett használható.**
Az engedélyezésért lépjen kapcsolatba az Insignia Health-szel az info@insigniahealth.com e-mail címen

# Patient Activation Measure® (PAM®) 13

Below are some statements that people sometimes make when they talk about their health. Please indicate how much you agree or disagree with each statement as it applies to you personally by circling your answer. There are no right or wrong answers, just what is true for you.

If the statement does not apply to you, circle N/A.

| | | | | | | |
|---|---|---|---|---|---|---|
| *1* | When all is said and done, I am the person who is responsible for taking care of my health | Disagree strongly | Disagree | Agree | Agree Strongly | NA |
| *2* | Taking an active role in my own health care is the most important thing that affects my health | Disagree strongly | Disagree | Agree | Agree Strongly | NA |
| *3* | I am confident I can help prevent or reduce problems associated with my health | Disagree strongly | Disagree | Agree | Agree Strongly | NA |
| *4* | I know what each of my prescribed medications do | Disagree strongly | Disagree | Agree | Agree Strongly | NA |
| *5* | I am confident that I can tell whether I need to go to the doctor or whether I can take care of the health problem myself | Disagree strongly | Disagree | Agree | Agree Strongly | NA |
| *6* | I am confident that I can tell a doctor concerns I have even when he or she does not ask | Disagree strongly | Disagree | Agree | Agree Strongly | NA |
| *7* | I am confident that I can follow through on medical treatments I may need to do at home | Disagree strongly | Disagree | Agree | Agree Strongly | NA |
| *8* | I understand my health problems and what causes them | Disagree strongly | Disagree | Agree | Agree Strongly | NA |
| *9* | I know what treatments are available for my health problems | Disagree strongly | Disagree | Agree | Agree Strongly | NA |
| *10* | I have been able to maintain (keep up with) lifestyle changes, like eating right or exercising | Disagree strongly | Disagree | Agree | Agree Strongly | NA |
| *11* | I know how to prevent problems with my health | Disagree strongly | Disagree | Agree | Agree Strongly | NA |
| *12* | I am confident I can figure out solutions when new problems arise with my health | Disagree strongly | Disagree | Agree | Agree Strongly | NA |
| *13* | I am confident that I can maintain lifestyle changes, like eating right and exercising even during times of stress | Disagree strongly | Disagree | Agree | Agree Strongly | NA |

Insignia Health, LLC®. Patient Activation Measure © 2003-2020 University of Oregon.
All rights reserved.

Contact Insignia Health at info@insigniahealth.com e-mail címen
