# Supplementary material for: Validation of the PAM-13 instrument in the Hungarian general population 40 years old and above
Source: Eur J Health Econ. 2022 Jan 31;23(8):1341–55. doi: 10.1007/s10198-022-01434-0 (PMC9550701; doi:10.1007/s10198-022-01434-0)
Supplement: Supplementary file 2 — Supplementary file2 (PDF 1081 KB) [file 10198_2022_1434_MOESM2_ESM.pdf]

Electronic Supplementary Material 2.

Zrubka Z, Vékás P, Németh P, Dobos Á, Hajdu O, Kovács L, Gulácsi L, Péntek M, *Validation of the PAM-13 instrument in the Hungarian general population*. European Journal of Health Economics 2021.

Sociodemographic characteristics of the survey respondents

		**Survey**		**Repeat Survey**		**General population 2011[a]**
		n	%	n	%	%
Total		900	-	100	-	-
Age group	40-49	173	*19*	16	*16*	*26*
	50-59	200	*22*	22	*22*	*28*
	60-69	348	*39*	38	*38*	*23*
	70+	149	*20*	24	*24*	*23*
Gender	Male	425	*47*	50	*50*	*44*
	Female	475	*53*	50	*50*	*56*
Education	Primary	251	*28*	47	*47*	*35*
	Secondary	326	*36*	32	*32*	*49*
	Tertiary	323	*36*	21	*21*	*16*
Region	Central Hungary	316	*35*	30	*30*	*29*
	Central Transdanubia	101	*11*	14	*14*	*11*
	Western Transdanubia	80	*9*	10	*10*	*10*
	Southern Transdanubia	96	*11*	10	*10*	*10*
	Northern Hungary	89	*10*	8	*8*	*12*
	Northern Great Plain	100	*11*	19	*19*	*15*
	Southern Great Plain	118	*13*	9	*9*	*13*
Type of settlement	Capital	209	*23*	22	*22*	*17*
	Town	513	*57*	49	*49*	*52*
	Village	178	*20*	29	*29*	*31*
Income	1st quintile	103	*13*	17	*19*	
	2nd quintile	119	*16*	17	*19*	
	3rd quintile	85	*11*	13	*14*	
	4th quintile	137	*18*	13	*14*	
	5th quintile	320	*42*	30	*33*	
	Missing	136	*15*	10	*10*	
Self-rated health	Very good	51	*6*	6	*6*	
	Good	305	*34*	32	*32*	
	Fair	449	*50*	47	*47*	
	Bad	79	*9*	13	*13*	
	Very Bad	13	*1*	1	*1*	
	Missing	3	*0*	1	*1*	
Chronic morbidity	No	309	*36*	29	*30*	
	Yes	560	*64*	68	*70*	
	Missing	31	*3*	3	*3*	
GALI	Not limited	579	*65*	53	*54*	
	Limited but not severely	274	*31*	43	*43*	
	Severely limited	43	*5*	3	*3*	
	Missing	4	*0*	1	*1*	
Exclusion	PAM-13 NA items >3	51	*6*	2	*2*	
	All PAM-13 items „disagree strongly”	18	*2*	4	*4*	
	All PAM-13 items „agree strongly”	23	*3*	5	*5*	
	PAM-13 completion time < 52s	7	*1*	0	*0*	
	Other reasons	22	*2*	4	*4*	
	Excluded in first administration	-	-	10	*10*	
