# Supplementary material for: Validation of the PAM-13 instrument in the Hungarian general population 40 years old and above
Source: Eur J Health Econ. 2022 Jan 31;23(8):1341–55. doi: 10.1007/s10198-022-01434-0 (PMC9550701; doi:10.1007/s10198-022-01434-0)
Supplement: Supplementary file 3 — Supplementary file3 (PDF 1081 KB) [file 10198_2022_1434_MOESM3_ESM.pdf]

Electronic Supplementary Material 3.

Zrubka Z, Vékás P, Németh P, Dobos Á, Hajdu O, Kovács L, Gulácsi L, Péntek M, *Validation of the PAM-13 instrument in the Hungarian general population*. European Journal of Health Economics 2021.

Health-related information seeking and online behaviours

Item 1: over the past 12 months how frequently have you sought for health-related information?

	**Low activity**		**High activity**	
	N	%	N	%
None	65	8.3	0	0.0
Few times past year	195	25.0	0	0.0
Bimonthly	53	6.8	0	0.0
Monthly	0	0.0	107	13.7
Several times per month	0	0.0	214	27.5
At least once a week	0	0.0	145	18.6
Total	313	40.2	466	59.8

Item 2: over the past 12 months how frequently have you participated in patient education or counseling about disease management or lifestyle?

	**Low activity**		**High activity**	
	N	%	N	%
None	608	*78.0*	0	*0.0*
Few times past year	0	*0.0*	119	*15.3*
Bimonthly	0	*0.0*	17	*2.2*
Monthly	0	*0.0*	17	*2.2*
Several times per month	0	*0.0*	12	*1.5*
At least once a week	0	*0.0*	6	*0.8*
Total	608	*78.0*	171	*22.0*

Item 3: over the past 12 months how frequently have you used the internet or a mobile device (such as smartphone, tablet or smart watch) for the following purposes: administrative chores concerning health or healthcare

	**Low activity**		**High activity**	
	N	%	N	%
None	336	*43.1*	0	*0.0*
Few times past year	0	*0.0*	222	*28.5*
Bimonthly	0	*0.0*	45	*5.8*
Monthly	0	*0.0*	89	*11.4*
Several times per month	0	*0.0*	69	*8.9*
At least once a week	0	*0.0*	18	*2.3*
Total	336	*43.1*	443	*56.9*

Item 4: over the past 12 months how frequently have you used the internet or a mobile device (such as smartphone, tablet or smart watch) for the following purposes: seeking for health related information

	**Low activity**		**High activity**	
	N	%	N	%
None	118	*15.1*	0	*0.0*
Few times past year	240	*30.8*	0	*0.0*
Bimonthly	0	*0.0*	56	*7.2*
Monthly	0	*0.0*	97	*12.5*
Several times per month	0	*0.0*	171	*22.0*
At least once a week	0	*0.0*	97	*12.5*
Total	358	*46.0*	421	*54.0*

Item 5: over the past 12 months how frequently have you used the internet or a mobile device (such as smartphone, tablet or smart watch) for the following purposes: health related communication with healthcare professionals, online helpers or peer patients

	**Low activity**		**High activity**	
	N	%	N	%
None	583	*74.8*	0	*0.0*
Few times past year	0	*0.0*	97	*12.5*
Bimonthly	0	*0.0*	15	*1.9*
Monthly	0	*0.0*	44	*5.6*
Several times per month	0	*0.0*	29	*3.7*
At least once a week	0	*0.0*	11	*1.4*
Total	583	*74.8*	196	*25.2*

Item 6: over the past 12 months how frequently have you used the internet or a mobile device (such as smartphone, tablet or smart watch) for the following purposes: any program supporting disease prevention or healthy lifestyle

	**Low activity**		**High activity**	
	N	%	N	%
None	499	*64.1*	0	*0.0*
Few times past year	0	*0.0*	115	*14.8*
Bimonthly	0	*0.0*	22	*2.8*
Monthly	0	*0.0*	52	*6.7*
Several times per month	0	*0.0*	51	*6.5*
At least once a week	0	*0.0*	40	*5.1*
Total	499	*64.1*	280	*35.9*

Item 7: over the past 12 months how frequently have you used the internet or a mobile device (such as smartphone, tablet or smart watch) for the following purposes: any program supporting the treatment, diagnosis, treatment or monitoring of specific disease condition?

	**Low activity**		**High activity**	
	N	%	N	%
None	432	*55.5*	0	*0.0*
Few times past year	0	*0.0*	184	*23.6*
Bimonthly	0	*0.0*	22	*2.8*
Monthly	0	*0.0*	44	*5.6*
Several times per month	0	*0.0*	66	*8.5*
At least once a week	0	*0.0*	31	*4.0*
Total	432	*55.5*	347	*44.5*
