# Supplementary material for: Validation of the PAM-13 instrument in the Hungarian general population 40 years old and above
Source: Eur J Health Econ. 2022 Jan 31;23(8):1341–55. doi: 10.1007/s10198-022-01434-0 (PMC9550701; doi:10.1007/s10198-022-01434-0)
Supplement: Supplementary file 4 — Supplementary file4 (PDF 1082 KB) [file 10198_2022_1434_MOESM4_ESM.pdf]

Electronic Supplementary Material 4.

Zrubka Z, Vékás P, Németh P, Dobos Á, Hajdu O, Kovács L, Gulácsi L, Péntek M, *Validation of the PAM-13 instrument in the Hungarian general population*. European Journal of Health Economics 2021.

Distribution of PAM-13 scores

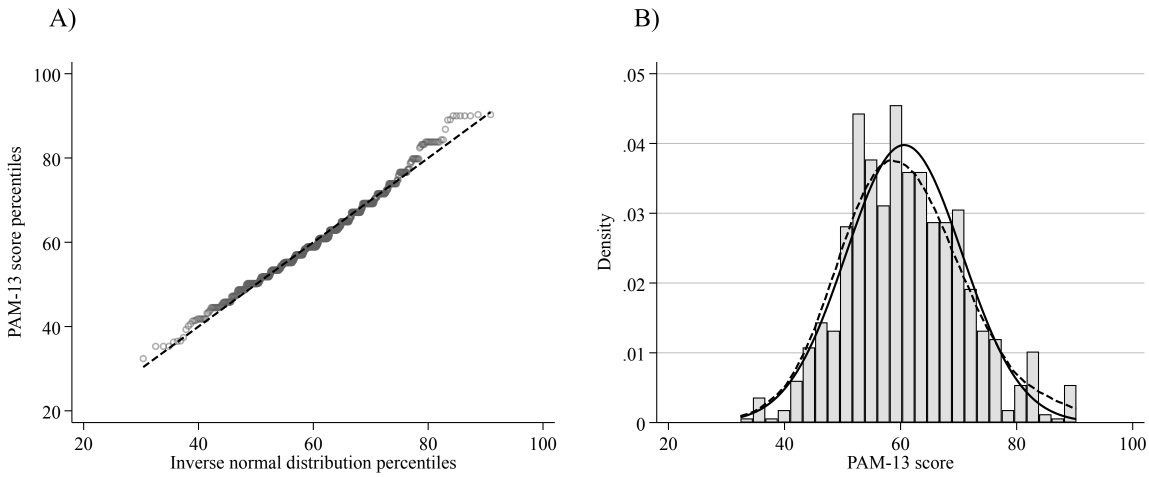

A) Quantile-plot vs normal distribution, B) histogram with kernel-density plot (dashed line) and normal curve (solid line)
