# Supplementary material for: Validation of the PAM-13 instrument in the Hungarian general population 40 years old and above
Source: Eur J Health Econ. 2022 Jan 31;23(8):1341–55. doi: 10.1007/s10198-022-01434-0 (PMC9550701; doi:10.1007/s10198-022-01434-0)
Supplement: Supplementary file 5 — Supplementary file5 (PDF 1081 KB) [file 10198_2022_1434_MOESM5_ESM.pdf]

Electronic Supplementary Material 5.

Zrubka Z, Vékás P, Németh P, Dobos Á, Hajdu O, Kovács L, Gulácsi L, Péntek M, *Validation of the PAM-13 instrument in the Hungarian general population*. European Journal of Health Economics 2021.

Bivariate hypothesis tests for known-groups validity

			**Subgroups**										
**Variable**	**Category**	Total	Male	Female	No chronic disease	Chronic disease	40-65 years old	65+ years old	2-5th Income quintile	1st Income quintile	Adequate health literacy[a]	Not adequate health literacy	Supported hypo-theses
Preventive behaviours	Preventive behaviour score ≥50%	61.09	59.86	62.04	63.68	60.30	61.59	61.02	61.09	60.96	60.43	62.30	
	Preventive behaviour score <50%	60.17	59.29	60.99	61.98	59.48	59.76	60.43	60.16	60.29	59.74	60.86	1 / 10
	p value	*0.102*	*0.288*	*0.149*	*0.090*	*0.185*	*0.022*	*0.316*	*0.104*	*0.402*	*0.215*	*0.121*	
Lifestyle risks stringent criteria	Lifestyle risk index=0	63.45	62.94	63.78	64.17	63.56	64.40	61.95	63.26	65.47	62.84	64.51	
	Lifestyle risk index ≥1	59.57	58.53	60.55	61.85	58.85	59.19	60.21	59.64	58.97	59.05	60.45	9 / 10
	p value	*<0.001*	*<0.001*	*0.002*	*0.040*	*<0.001*	*<0.001*	*0.109*	*<0.001*	*0.013*	*<0.001*	*0.002*	
Lifestyle risks relaxed criteria	Lifestyle risk index ≤1	62.30	60.81	63.40	63.62	61.93	62.93	61.36	62.20	63.28	61.66	63.43	
	Lifestyle risk index ≥2	57.84	57.86	57.81	60.31	57.11	57.00	59.41	58.02	56.17	57.41	58.53	9 / 10
	p value	*<0.001*	*0.002*	*<0.001*	*0.005*	*<0.001*	*<0.001*	*0.056*	*<0.001*	*0.006*	*<0.001*	*<0.001*	
Health information seeking	At least monthly	61.58	61.13	61.88	63.48	61.13	61.85	61.17	61.53	62.02	60.70	63.05	
	Less often than monthly	59.18	57.89	60.80	61.62	57.96	58.76	59.92	59.36	56.71	59.19	59.16	7 / 9
	p value	*<0.001*	*0.001*	*0.158*	*0.065*	*<0.001*	*<0.001*	*0.153*	*0.002*	*0.041*	*0.045*	*0.001*	
Patient education	Over past year	62.09	60.29	63.14	65.25	61.40	62.51	61.35	61.94	63.26	61.57	62.87	
	None	60.20	59.40	60.97	62.20	59.41	60.01	60.52	60.27	59.61	59.69	61.11	6 / 9
	p value	*0.018*	*0.266*	*0.031*	*0.047*	*0.030*	*0.013*	*0.290*	*0.035*	*0.139*	*0.044*	*0.124*	
Online health information seeking	At least bimonthly	61.04	60.41	61.67	64.05	60.16	61.28	60.79	61.03	61.13	60.27	62.45	
	Less often than bimonthly	60.13	58.84	61.43	61.53	59.60	59.68	60.60	60.17	59.73	59.85	60.55	2 / 9
	p value	*0.104*	*0.062*	*0.409*	*0.018*	*0.271*	*0.039*	*0.437*	*0.125*	*0.306*	*0.316*	*0.062*	
Online health-related communication	Over past year	61.83	61.26	62.18	63.95	61.45	61.79	61.87	61.81	61.95	61.44	62.32	
	None	60.21	59.10	61.26	62.36	59.29	60.20	60.23	60.23	60.08	59.70	61.19	4 / 9
	p value	*0.025*	*0.040*	*0.205*	*0.160*	*0.014*	*0.063*	*0.114*	*0.033*	*0.263*	*0.046*	*0.203*	
Online health prevention activity	Over past year	61.64	60.68	62.23	62.99	61.42	62.21	60.76	61.45	63.51	61.04	62.40	
	None	60.04	59.08	61.03	62.44	59.11	59.68	60.65	60.16	58.95	59.64	60.86	3 / 9
	p value	*0.015*	*0.068*	*0.119*	*0.328*	*0.006*	*0.003*	*0.465*	*0.045*	*0.058*	*0.069*	*0.105*	
Online disease management activity	Over past year	61.43	59.94	62.32	64.00	60.90	62.24	61.03	61.23	63.06	60.74	62.47	
	None	59.97	59.33	60.68	61.90	58.99	59.42	60.36	60.15	58.07	59.60	60.66	4 / 9
	p value	*0.022*	*0.278*	*0.051*	*0.049*	*0.017*	*0.001*	*0.289*	*0.075*	*0.030*	*0.103*	*0.070*	
	N	779	358	421	253	503	483	296	704	75	491	288	
	Supported hypotheses	7 / 9	4 / 9	3 / 9	5 / 9	7 / 9	8 / 9	0 / 9	6 / 9	4 / 9	5 / 9	3 / 9	

[a] NVS
