# Supplementary material for: Validation of the PAM-13 instrument in the Hungarian general population 40 years old and above
Source: Eur J Health Econ. 2022 Jan 31;23(8):1341–55. doi: 10.1007/s10198-022-01434-0 (PMC9550701; doi:10.1007/s10198-022-01434-0)
Supplement: Supplementary file 6 — Supplementary file6 (PDF 1081 KB) [file 10198_2022_1434_MOESM6_ESM.pdf]

Electronic Supplementary Material 6.

Zrubka Z, Vékás P, Németh P, Dobos Á, Hajdu O, Kovács L, Gulácsi L, Péntek M, *Validation of the PAM-13 instrument in the Hungarian general population*. European Journal of Health Economics 2021.

Regression analyses using PAM-13 scores as predictor

		PBS[i]	LRI[j]	BMI[k]	Smoking[l]	Alcohol[m]	Physical activity[n]	Diet[o]	HIS[p]	PPE[q]	OHA[r]	OHIS[s]	OHC[t]	OHP[u]	ODA[v]
Model		OLS	Robust regression	Logistic	Logistic	Logistic	Logistic	Logistic	Ordered logit	Ordered logit	Ordered logit	Ordered logit	Ordered logit	Ordered logit	Ordered logit
PAM-13 score		0.00	-0.02***	-0.05***	0.00	-0.01	-0.04***	-0.08**	0.00	0.01	0.00	-0.01	0.00	0.00	-0.01
eHEALS[a]		0.00	0.00	0.03	-0.01	0.01	-0.01	-0.05	0.16***	0.04	0.05**	0.14***	0.07***	0.09***	0.11***
NVS[b]		0.00	-0.04	0.00	-0.09	-0.12	0.00	-0.16	0.00	-0.14*	-0.07	-0.02	-0.20***	-0.17**	-0.09
Age		0.00	-0.01*	0.01	-0.04***	0.00	-0.01	-0.04*	0.01	-0.02	0.01	-0.01	0.01	0.00	0.02**
Gender		-0.01	-0.09	-0.11	0.07	-0.82***	0.23	-0.43	0.65***	0.41*	0.23	0.61***	0.35	0.39*	0.46**
Education[c]	Secondary	0.05*	-0.03	-0.01	-0.56*	0.27	0.23	0.05	-0.03	-0.05	-0.31	-0.09	-0.38	-0.11	-0.13
	Tertiary	0.10***	-0.20	-0.24	-0.99***	0.37	0.08	-1.03	0.35	0.39	0.28	0.53*	0.40	0.15	0.16
Income[d]	2nd quintile	0.00	0.16	0.00	0.40	-0.38	0.76*	-0.32	-0.51	0.06	-0.36	-0.18	0.13	0.19	-0.43
	3rd quintile	0.00	0.03	-0.34	0.38	-0.34	0.42	-0.11	-0.63*	-0.25	-0.53	-0.51	0.16	0.13	-0.64
	4th quintile	0.06	-0.08	-0.14	0.10	-0.53	0.24	-0.94	-0.24	-0.09	-0.17	0.10	0.55	0.26	-0.04
	5th quintile	0.04	0.16	0.13	0.61	-0.16	0.47	-0.62	-0.36	-0.07	-0.30	-0.34	0.03	-0.07	-0.63*
Chronic morbidity[e]	Yes	0.05*	0.13	0.59**	-0.13	-0.79**	0.55*	0.02	-0.08	0.65*	0.51**	0.14	0.58*	-0.14	0.39
Self-rated health[f]	Bad	-0.01	0.50	2.26*	0.36	-1.22	0.02	12.39	-0.67	-0.35	-0.86	-0.14	-1.25	-1.50*	-1.13
	Fair	-0.04	0.36	1.91	0.11	-0.22	0.09	10.91	-1.43*	-0.78	-1.22	-0.66	-0.90	-1.52*	-0.99
	Good	-0.06	0.11	1.69	-0.06	-1.13	-0.21	11.01	-1.83*	-1.08	-1.38*	-0.93	-0.91	-1.26	-0.97
	Very good	-0.04	-0.12	1.33	-2.04	-2.59	0.39	11.00	-1.68*	-0.72	-2.32**	-1.36	-0.77	-1.36	-0.92
GALI[g] (Limited due to	Not severely	0.03	-0.01	-0.36	-0.12	0.14	0.36	-0.21	0.58**	0.17	0.23	0.65***	0.79***	0.34	0.17
health problems)	Severely	0.10	-0.25	-0.28	-0.17	-1.03	0.31	-15.48	0.39	0.35	0.92*	0.67	0.62	0.63	0.16
Settlement[h]	Town	-0.04	-0.06	0.08	-0.29	0.48	-0.38	-0.14	0.16	0.03	-0.19	0.07	-0.17	-0.36	-0.06
	Village	-0.09**	-0.02	0.26	-0.16	0.41	-0.44	0.09	-0.14	-0.85*	-0.54*	-0.37	-0.54	-0.46	-0.31
Constant		0.38**	3.07***	-1.11	2.37	0.45	1.9	-4.32							
N		648	648	648	648	648	648	648	648	648	648	648	648	648	648
Breusch-Pagan test	*p* value	0.214	-	-	-	-	-	-	-	-	-	-	-	-	-
Ramsey RESET test	*p* value	0.188	0.705	-	-	-	-	-	-	-	-	-	-	-	-
Goodness of Fit test	*p* value	-	-	0.32	0.019	0.344	0.256	0.997	0.284	0.928	0.329	0.12	0.412	0.176	0.728

*p < 0.05 ; **p<0.01; ***p<0.001

[a] eHealth Literacy Scale;[b]Newest Vital Sign; [c]base: Primary; [d]base: 1st quintile ; [e]base: No chronic morbidity; [f]base: Very bad, [g]Global activity limitation indicator, base: no limitation; [h]base: Capital; [i]Preventive behaviour score; [j]Lifestyle risk index; [k]18.5<BMI(body mass index)<30; [l]Current smoker; [m]Binge drinking ≥1 per week; [n]Sedentary behaviour ≥8 hours per day with <150 min exercise per week or no exercise at all; [o]no fruit and / or vegetable intake; [p]Health information seeking (general); [q]Participation in patient education;[r]Online health administration; [s]Online health information seeking; [t]Online health-related communication; [u]Online health prevention; [v]Online disease management activity
