# Supplementary material for: Validation of the PAM-13 instrument in the Hungarian general population 40 years old and above
Source: Eur J Health Econ. 2022 Jan 31;23(8):1341–55. doi: 10.1007/s10198-022-01434-0 (PMC9550701; doi:10.1007/s10198-022-01434-0)
Supplement: Supplementary file 7 — Supplementary file7 (PDF 1081 KB) [file 10198_2022_1434_MOESM7_ESM.pdf]

Electronic Supplementary Material 7.

Zrubka Z, Vékás P, Németh P, Dobos Á, Hajdu O, Kovács L, Gulácsi L, Péntek M, *Validation of the PAM-13 instrument in the Hungarian general population*. European Journal of Health Economics 2021.

Regression analyses using PAM-13 levels as predictor

		PBS[i]	LRI[j]	BMI[k]	Smoking[l]	Alcohol[m]	Physical activity[n]	Diet[o]	HIS[p]	PPE[q]	OHA[r]	OHIS[s]	OHC[t]	OHP[u]	ODA[v]
Model		OLS	Robust regression	Logistic	Logistic	Logistic	Logistic	Logistic	Ordered logit	Ordered logit	Ordered logit	Ordered logit	Ordered logit	Ordered logit	Ordered logit
PAM level	2	0.07	-0.30	-0.67	-0.31	0.08	-0.42	0.34	-0.05	-0.74	0.21	-0.40	0.43	0.53	0.29
	3	0.06	-0.56***	-0.95**	-0.21	-0.08	-0.93**	-0.77	-0.13	-0.34	0.01	-0.47	0.24	0.55	0.17
	4	0.04	-0.75***	-1.92***	0.32	-0.39	-1.07*	-17.18	-0.13	-0.05	-0.02	-0.55	0.04	0.06	-0.18
eHEALS[a]		0.00	0.00	0.02	-0.02	0.00	-0.02	-0.06	0.16***	0.04*	0.05**	0.14***	0.08***	0.09***	0.11***
NVS[b]		0.00	-0.04	0.00	-0.09	-0.12	0.00	-0.17	0.00	-0.14*	-0.07	-0.01	-0.21***	-0.16**	-0.09
Age		0.00	-0.01*	0.01	-0.04***	0.00	-0.01	-0.04*	0.01	-0.02	0.01	-0.01	0.01	0.00	0.02**
Gender		-0.01	-0.10	-0.13	0.07	-0.82***	0.20	-0.51	0.66***	0.44*	0.22	0.61***	0.35	0.39*	0.46**
Education[c]	Secondary	0.06*	-0.03	-0.01	-0.58*	0.28	0.23	0.14	-0.03	-0.12	-0.30	-0.10	-0.33	-0.08	-0.11
	Tertiary	0.10***	-0.19	-0.23	-0.98***	0.36	0.11	-1.12	0.36	0.37	0.29	0.54*	0.43	0.13	0.16
Income[d]	2nd quintile	-0.01	0.18	-0.02	0.49	-0.40	0.79*	-0.60	-0.51	0.13	-0.37	-0.15	0.09	0.10	-0.47
	3rd quintile	0.00	0.04	-0.35	0.41	-0.34	0.43	-0.19	-0.63*	-0.19	-0.54	-0.50	0.11	0.07	-0.66
	4th quintile	0.06	-0.05	-0.15	0.17	-0.53	0.30	-1.03	-0.23	-0.05	-0.17	0.11	0.51	0.18	-0.07
	5th quintile	0.03	0.18	0.10	0.67	-0.18	0.49	-0.73	-0.36	0.01	-0.31	-0.33	-0.03	-0.16	-0.67*
Chronic morbidity[e]	Yes	0.05*	0.12	0.61**	-0.13	-0.80**	0.53*	-0.01	-0.09	0.68*	0.50**	0.13	0.56*	-0.12	0.40
Self-rated health[f]	Bad	-0.01	0.49	2.22	0.41	-1.23	0.03	13.96	-0.66	-0.37	-0.87	-0.15	-1.22	-1.53*	-1.15
	Fair	-0.04	0.36	1.84	0.19	-0.26	0.11	12.35	-1.42*	-0.75	-1.23	-0.67	-0.90	-1.63*	-1.05
	Good	-0.07	0.08	1.58	0.01	-1.19	-0.24	12.28	-1.82*	-1.03	-1.40*	-0.95	-0.90	-1.38	-1.05
	Very good	-0.04	-0.18	1.23	-2.04	-2.64	0.26	12.39	-1.67*	-0.67	-2.34**	-1.39	-0.74	-1.45	-0.98
GALI[g] (Limited due to	Not severely	0.03	-0.01	-0.37	-0.09	0.13	0.36	-0.25	0.58**	0.18	0.23	0.67***	0.78***	0.32	0.17
health problems)	Severely	0.09	-0.24	-0.36	-0.09	-1.06	0.33	-17.65	0.40	0.38	0.92*	0.69	0.60	0.55	0.11
Settlement[h]	Town	-0.04	-0.07	0.07	-0.30	0.48	-0.40	-0.09	0.16	0.05	-0.19	0.06	-0.17	-0.36	-0.06
	Village	-0.09**	-0.03	0.23	-0.18	0.40	-0.45	0.30	-0.14	-0.86*	-0.52*	-0.38	-0.52	-0.48	-0.31
Constant		0.36**	2.36***	-2.57	2.77*	-0.04	0.64	-9.22							
N		648	648	648	648	648	648	612	648	648	648	648	648	648	648
Breusch-Pagan test	*p* value	0.431	-	-	-	-	-	-	-	-	-	-	-	-	-
Ramsey RESET test	*p* value	0.308	0.734	-	-	-	-	-	-	-	-	-	-	-	-
Goodness of Fit test	*p* value	-	-	0.307	0.031	0.342	0.261	1	0.314	0.523	0.199	0.014	0.366	0.825	0.691

*p < 0.05 ; **p<0.01; ***p<0.001

[a] eHealth Literacy Scale;[b]Newest Vital Sign; [c]base: Primary; [d]base: 1st quintile ; [e]base: No chronic morbidity; [f]base: Very bad, [g]Global activity limitation indicator, base: no limitation; [h]base: Capital; [i]Preventive behaviour score; [j]Lifestyle risk index; [k]18.5<BMI(body mass index)<30; [l]Current smoker; [m]Binge drinking ≥1 per week; [n]Sedentary behaviour ≥8 hours per day with <150 min exercise per week or no exercise at all; [o]no fruit and / or vegetable intake; [p]Health information seeking (general); [q]Participation in patient education;[r]Online health administration; [s]Online health information seeking; [t]Online health-related communication; [u]Online health prevention; [v]Online disease management activity
