# Supplementary material for: Validation of the PAM-13 instrument in the Hungarian general population 40 years old and above
Source: Eur J Health Econ. 2022 Jan 31;23(8):1341–55. doi: 10.1007/s10198-022-01434-0 (PMC9550701; doi:10.1007/s10198-022-01434-0)
Supplement: Supplementary file 10 — Supplementary file10 (PDF 1081 KB) [file 10198_2022_1434_MOESM10_ESM.pdf]

Electronic Supplementary Material 10.

Zrubka Z, Vékás P, Németh P, Dobos Á, Hajdu O, Kovács L, Gulácsi L, Péntek M, *Validation of the PAM-13 instrument in the Hungarian general population*. European Journal of Health Economics 2021.

Adjusted probability of lifestyle-related risks at various PAM-13 levels in the 65+ subgroup

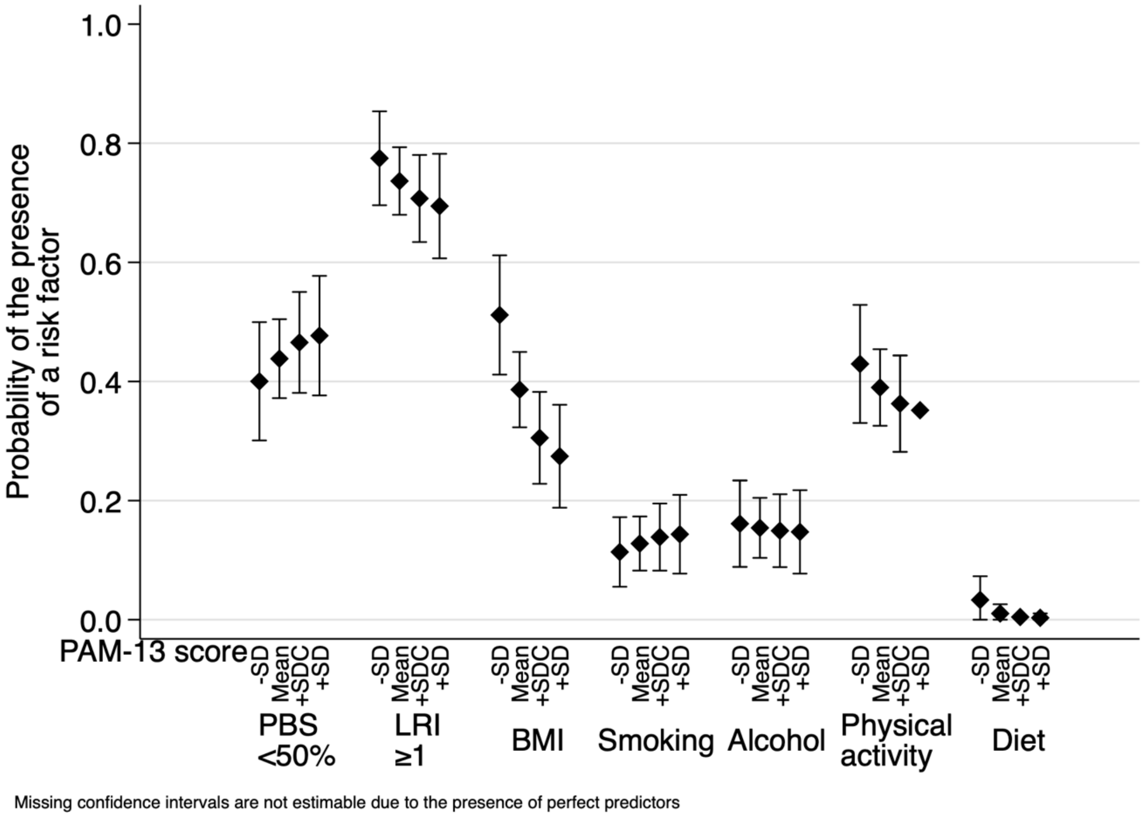

BMI: body mass index; PBS: prevention behaviour score; LRI: lifestyle risk index; +/- SD: mean +/- 1 standard deviation; +SDC: mean + smallest detectable change
